# Effect of Gold Nanoparticle Distribution in TiO_2_ on the Optical and Electrical Characteristics of Dye-Sensitized Solar Cells

**DOI:** 10.1186/s11671-017-2285-4

**Published:** 2017-08-29

**Authors:** Shuichi Mayumi, Yutaka Ikeguchi, Daisuke Nakane, Yasuaki Ishikawa, Yukiharu Uraoka, Mamoru Ikeguchi

**Affiliations:** 1 PGS Home Co., Ltd., 2-1-8, Higashiimazato, Higashinariku, Osaka, 537-0011 Japan; 2 Graduate School of Materials Science, Nara Institute of Science and Technology, 8916-5 Takayama-cho, Ikoma, Nara 630-0192 Japan

**Keywords:** Dye-sensitized solar cells, Gold nanoparticles, Plasmonics, Optical properties, Titanium dioxide

## Abstract

Photoanodes comprising Au nanoparticles (GNPs) and thin TiO_2_ layers with a stacked structure were fabricated by repeating the application of TiO_2_ paste and GNP solutions on conductive glass to vary the distribution of GNPs in the TiO_2_ layer. The plasmon-enhanced characteristics of dye-sensitized solar cells (DSSCs) with such photoanodes were investigated. Both the absorption of the TiO_2_ layer and the performance of the DSSC are found to be most increased by plasmonic enhancement when GNPs are concentrated near the position in the TiO_2_ layer, which is the penetration depth of the incident light of wavelength corresponding to the maximum absorption of the N719 dye (~ 520 nm). When a GNP layer with a relatively high density of 1.3 μg/cm^2^ density was formed at its position, and two GNP layers with a relatively low density of 0.65 μg/cm^2^ were formed near the front side of the incident light, the short-circuit current density (*J*sc) and energy conversion efficiency (*η*) of the DSSC were found to be 10.8 mA/cm^2^ and 5.0%, increases of 15 and 11%, respectively, compared with those of the DSSC without GNPs. Our work suggests that optimization of the distribution of GNPs in the TiO_2_ layer is very important for improving the performance of DSSCs fabricated by utilizing GNPs.

**Electronic supplementary material:**

The online version of this article (10.1186/s11671-017-2285-4) contains supplementary material, which is available to authorized users.

## Background

S﻿ince their development in 1991 by O’Regan and Grätzel [1], dye-sensitized solar cells (DSSCs) have attracted much attention because of their simple fabrication process, potential for low-cost production, and mild impact on the environment [2–4]. However, the energy conversion efficiencies of DSSCs are not yet high enough for practical use and are lower than those of other technologies such as perovskite-sensitized solar cells [5], thin-film solar cells [6], and crystalline silicon solar cells [7]. One approach to increase the efficiency of DSSCs is to enhance the light absorption. Increasing the thickness of the TiO_2_ layer in DSSCs enhances the light absorption due to the increase in the number of dye molecules adsorbed on the TiO_2_ for light harvesting. However, this approach may lower the efficiency due to the recombination of photoelectrons that have to travel a longer distance to reach the collecting electrode [8]. The technology of nanophotonics for light management inside the solar cell has been suggested as another approach to achieve high efficiencies [9, 10]. Metal nanoparticles can contribute to effective light absorption in solar cells, both by local-field enhancement through localized surface plasmon resonance and by light scattering leading to prolonged optical path lengths. Au and Ag are mainly employed as nanoparticles in DSSCs because their surface plasmon resonance can be tuned in the visible wavelength region where common synthetic dyes mostly absorb [11–14]. Au nanoparticles (GNPs) are generally applied in the TiO_2_ layer by blending with TiO_2_ nanopowder, which is then used to fabricate conformal TiO_2_-Au nanocomposite films [15–17]. SiO_2_-coated Au nanoparticles and TiO_2_-coated Ag nanoparticles have also been applied to DSSCs [18–21]. A method of forming Ag nanoparticles on the both top and bottom surfaces of a TiO_2_ layer by utilizing sputtering and annealing has been published [22]. GNPs synthesized by physical vapor deposition have also been reported to enhance photocurrents in DSSCs [23]. In addition, a method of using a tailored bimodal size distribution of functionalized GNPs that have been chemically immobilized onto a TiO_2_ layer via dithiodibutyric acid linkers has been published [24]. However, to our knowledge, an effective approach to vary the distribution of metal nanoparticles in the TiO_2_ layer to improve the performance of DSSCs has not yet been published. It is important to optimize the distribution of expensive metal nanoparticles such as Au or Ag in TiO_2_ layers to enhance the efficiency at relatively low cost. In this work, we have studied the correlation between the distributions of GNPs in a TiO_2_ layer and the optical absorption characteristics of the TiO_2_ layer to obtain an optimum distribution of GNPs for improving the performance of DSSCs. The distribution of GNPs in the TiO_2_ layer was adjusted by repeating the process of applying TiO_2_ paste and GNP solutions with a controlled quantity of GNPs on the conductive glass, forming a stacked structure comprising GNPs and thin TiO_2_ layers.

## Methods

### Materials

DSSCs were fabricated using the following materials: glass substrate coated with indium tin oxide (ITO) transparent conductive oxide (TCO) film with a sheet resistance of approximately 10 Ω sq^− 1^ (no. 0052; Geomatec Co., Ltd.), iodine, 1, 2-dimethyl-3-propyl imidazolium iodide (DMPII), and acetonitrile (Tokyo Chemical Industry Co., Ltd., Japan), anhydrous lithium iodide (Wako Pure Chemical Industries, Ltd.), hydrogen tetrachloroaurate(III) trihydrate and di-tetrabutylammonium *cis*-bis (isothiocyanato) bis (2, 2′-bipyridyl-4, 4′-dicarboxylato) ruthenium (II) (N719), 4-tert-butylpyridine (TBP) and chloroplatinic acid hexahydrate (Sigma-Aldrich), titanium oxide paste with a particle size of approximately 20 nm (PST-18NR, JGC Catalysts and Chemicals Ltd), Himilan films with a thickness of 50 μm (Peccell Technologies, Inc., Japan), and cover glass with a diameter of 12 mm (Fisher). The above ITO-based TCO 0052 is heat resistant, unlike conventional ITO-based TCO. The substrate was also utilized in Ref [25], and its optical and electrical characteristics were not deteriorated even after annealing at temperatures as high as 500 °C.

### Synthesis of Gold Nanoparticles

GNPs were synthesized using the well-known Turkevich method [26]. A 100 ml solution of 0.01 wt% hydrogen tetrachloroaurate (III) trihydrate in deionized water was heated until boiling on a hot plate. Next, 3.5 ml of 1 wt% trisodium citrate dihydrate aqueous solution was added to the boiling solution under vigorous stirring. The solution was kept boiling and stirring for 60 min. With this method, GNPs of ~ 20 nm were obtained. To obtain GNPs of ~ 40 nm, 6 ml of the solution with GNPs of ~ 20 nm was added as seeds to a 100 ml solution of 0.01 wt% hydrogen tetrachloroaurate(III) trihydrate in boiled deionized water, followed by adding 0.5 ml of 1 wt% trisodium citrate dihydrate aqueous solution. Seed particles with sizes of ~ 40 and ~ 60 nm were used to obtain GNPs of ~ 60 and ~ 90 nm, respectively. After the synthesis of GNPs was completed, the solution was centrifuged at 10,000 rpm for 20 min. After the supernatant was removed, the GNPs collected from the bottom of tubes were dispersed in a mixture of deionized water and ethanol with a ratio of 1/10 in volume, forming a GNP solution to be used in DSSC fabrication. The Stöber method was used to coat ~ 20 nm GNPs with SiO_2_ films [27, 28]. 0.6 ml of 112 mM tetraethyl orthosilicate and 0.09 ml of ammonium solution were added to 2.5 ml of propanol containing 0.5 ml of GNP water solution under vigorous stirring. The stirring was maintained for 15 min, and SiO_2_ films with a thickness of ~20 nm were formed.

### Fabrication of Photoanodes and Assembly of DSSCs

The photoanodes with a stacked structure of GNPs and TiO_2_ layers were fabricated by repeating the formation of a thin TiO_2_ layer and a GNP layer. The TiO_2_ paste was coated on TCO-coated glass by a screen-printing method and then annealed at 450 °C for 15 min. The thickness of each thin TiO_2_ layer was ~ 1.1 μm after the annealing. The approximate area of the prepared porous TiO_2_ layer was 25 mm^2^ (5 mm × 5 mm). The GNP solution was applied on the surface of the annealed TiO_2_ layer by drop casting and natural drying. The density of GNPs in the TiO_2_ layer was varied by changing the quantity or the GNP concentration of the applied GNP solution. The concentration in GNPs of the solution was calculated by measuring the weight of GNPs in a certain volume of the solution. A stacked structure of GNP and TiO_2_ layers was formed by repeating the formation of TiO_2_ and GNP layers. Final annealing of the TiO_2_ layer was performed at 500 °C for 30 min. Dye adsorption was carried out by immersing the TiO_2_ electrode in a 0.3 mM ethanol solution of N 719 at 25 °C for 20 h. To prepare the counter electrode, a few drops of 2 mg chloroplatinic acid hexahydrate in 1 ml ethanol solution were placed on TCO-coated glass drilled with a 0.9-mm-diameter hole. The counter electrode was heated at 400 °C for 30 min. The fabrication process of a typical sandwich-type DSSC was as follows. The counter electrode and the dye-sensitized photoanode were sandwiched with a Himilan film as a spacer and were then joined together by melting the film on a hotplate to form an open cell. An electrolyte containing 0.05 M I_2_, 0.05 M LiI, 0.6 M DMPII, and 0.5 M TBP in acetonitrile was injected into the open cell through the hole in the counter electrode and was filled in a vacuum chamber. Finally, the hole was sealed by melting a Himilan film lying between the counter electrode and a cover glass on a hotplate.

### Characterizations

The absorption spectra of GNPs dispersed in water were measured using a UV/Visible Spectrophotometer (Amersham Biosciences Ultrospec 3300 pro). The GNPs were observed using a transmission electron microscope (TEM, JEM-2200FS, JEOL). The surface morphologies of the GNPs–TiO_2_ photoanodes were examined with a scanning electron microscope (SEM, SU6600, Hitachi). The thickness of the TiO_2_ layer was measured by a surface profiler (AS500, KLA Tencor). The current density–voltage (*J*–*V*) characteristics and the incident photon-to-current efficiency (IPCE) spectra of the fabricated DSSCs and optical absorption spectra of the photoanodes were measured using spectral sensitivity measuring equipment (CEP-2000, BUNKOUKEIKI), which irradiated light at 100 mW cm^− 2^ (AM 1.5). The effective irradiated area of each cell was kept as 0.05 cm^2^ by using a light-tight metal mask for all samples.

## Results and Discussion

### Morphologies and Optical Properties of Au Nanoparticles

**Fig. 1:**
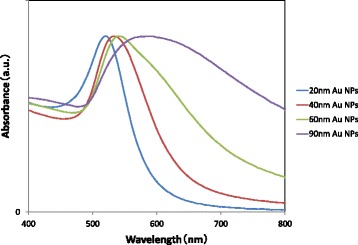
Absorbance spectra of GNPs of various sizes

**Fig. 2:**
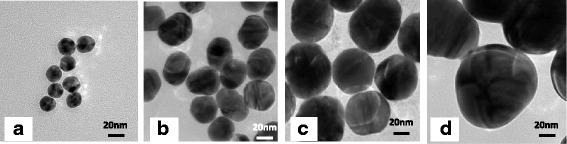
TEM images of the **a** ~ 20, **b** ~ 40, **c** ~ 60, and **d** ~ 90 nm GNPs

Figure 1 shows the absorption spectra of GNPs of various sizes dispersed in water. The TEM images of GNPs used in the present work are shown in Fig. 2, which indicates that the GNPs are mono-dispersed with a spherical morphology. A red shift in the resonance–wavelength was observed with increasing size of GMPs due to electromagnetic retardation in larger particles, which is in accordance with the reported literature [17, 29–31]. The size of GNPs was determined by comparing the absorption spectra of the as-prepared samples with the data available in the literature. As the size of GNPs increases, the absorption spectrum exhibits a broad feature in the red region due to the presence of bigger particles formed possibly by aggregation during their synthesis [17]. This tendency is remarkable for GNPs with sizes more than ~ 60 nm. It was also confirmed by TEM observation that the size distribution became very large when the GNPs became larger than 60 nm.

**Fig. 3:**
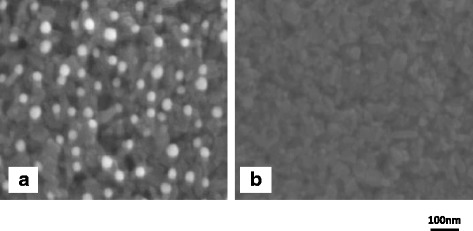
SEM images of the surfaces of TiO_2_ layers **a** with and **b** without GNPs. GNPs were formed by dropping the solution containing ~ 40 nm GNPs on the surfaces of TiO_2_ layers and drying

Figure 3a shows a typical SEM image of ~ 40 nm GNPs formed by applying and drying a GNP solution on the surface of the TiO_2_ layer. A SEM image of the surface of the TiO_2_ layer without GNPs is shown in Fig. 3b for comparison. It is obvious that most of the GNPs disperse on the surface of the TiO_2_ layers almost uniformly with very few aggregations. The aggregations tended to increase with an increase in the density of GNPs. Presumably, GNPs aggregate during the drying of the nanoparticle solution applied to the substrate. Also, in the case of GNPs of sizes other than ~ 40 nm, uniform dispersion of GNPs on TiO_2_ layers was observed with an SEM, suggesting that the method of application and drying of GNP solutions is effective in forming GNP layers in the TiO_2_ layers.

### Size Effects of Au Nanoparticles on DSSC Performance

The photovoltaic performances of DSSCs with GNPs of different sizes are listed in Table 1.

**Table 1:** Photovoltaic properties of DSSCs with and without GNPs of various sizes

Sample details	*J*sc(mA/cm^2^)	*V*oc (V)	FF	*η* (%)
Reference DSSC (without Au nanoparticles)	2.20	0.74	0.75	1.2
Au 20 nm	2.80	0.70	0.65	1.3
Au 20 nm/SiO_2_ 20 nm	2.74	0.73	0.73	1.5
Au 40 nm	2.99	0.73	0.75	1.6
Au 60 nm	3.18	0.74	0.75	1.8
Au 90 nm	2.69	0.74	0.75	1.5

In this case, the GNPs were formed between the conductive glasses and very thin TiO_2_ layers of 1.3 μm thickness by dropping GNP solutions on the surface of the conductive glass and drying naturally. The weight density of GNPs applied for all samples was the same (1.3 μg/cm^2^). Short-circuit current density (*J*sc) and energy conversion efficiency (*η*) are found to increase by applying GNPs of any size, compared with those of DSSCs without GNPs. Such an increase in *J*sc is caused by the plasmonic effect of GNPs, which has also been demonstrated in previous studies [15–17]. *J*sc and *η* are found to increase upon increasing GNP size from ~ 20 to ~ 60 nm and decrease upon increasing GNP size from ~ 60 to ~ 90 nm. The largest increases in Jsc and *η* of ~ 45% by the application of ~ 60 nm GNPs were obtained without changes in open-circuit voltage (*V*oc) and fill factor (FF). On the other hand, decreases in *V*oc and FF were observed for DSSCs with smaller GNPs of ~ 20 nm size. The decrease in *V*oc may be attributed to an increase in backward charge transfer from the TiO_2_ to the electrolyte due to exposed GNPs since ~ 20 nm GNPs covered with ~ 20-nm thick SiO_2_ films did not cause such a decrease in *V*oc. The SiO_2_ films act as an insulator to inhibit charge recombination on the metal surface [21]. At this stage, the reason why *V*oc decreased only in the case of smaller GNPs cannot be explained clearly. However, it is speculated that the total surface area of GNPs acting as recombination centers may be larger for smaller particles, as the weight density of GNPs applied for all samples was the same value (1.3 μg/cm^2^).

For ~ 20 nm GNPs, the coating process of GNPs with SiO_2_ films is necessary to observe plasmonic enhancement in this study. Conversely, for large GNPs above ~ 60 nm, repeating the process of GNPs synthesis is necessary and the variation in size of GNPs may increase due to aggregation of GNPs, thus lowing experimental accuracy. Therefore, for most investigations in this study, we employed ~ 40 nm GNPs, which have relatively small variations in size and show sufficiently large increases in *J*sc and *η* (~ 36 and ~ 33%, respectively) compared with DSSCs without GNP.

### Correlation of the Optical Absorption Characteristics of the TiO_2_ Layer and the Performance of DSSCs with the Position of the Au Nanoparticle Layer in the TiO_2_ Layer

**Fig. 4:**
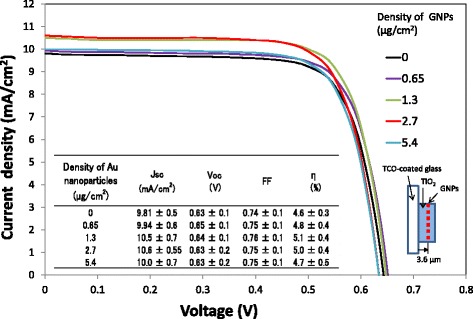
Current density–voltage curves of the DSSCs with changing the density of GNPs per GNP layer. Four cells for each density of GNPs were fabricated. The GNP layer is formed in a TiO_2_ layer of 6.0 μm thickness at the position of 3.6 μm from the TCO surface

Before studying the correlation between the position of a GNP layer in TiO_2_ film and the performance of the DSSCs, the optimum quantity of GNPs per GNP layer was investigated to obtain high plasmonic enhancement effects. Current density–voltage curves of the DSSCs with changing the density of ~ 40 nm GNPs per GNP layer are shown in Fig. 4. The density of GNPs was changed by varying the quantity of the GNP solution. The GNP layer was formed at a position of 3.6 μm from the surface of the conductive glass in TiO_2_ layers of 6.0 μm thickness. Obviously, as the density of GNPs increases from 0 to 1.3 or 2.7 μg/cm^2^, *J*sc and *η* increase due to the plasmon enhancement by the GNPs. However, when the density of GNPs increases up to 5.4 μg/cm^2^_,_ *J*sc and *η* decrease because excess GNPs aggregate, diminish the localized plasmonic effect, and block incident light. Actually, as the quantity of the GNP solution used for coating increased, it was visually observed that the photoanode took on the color of the metal and became cloudy. It should be noted that in Fig. 4, the deviations in *J*sc and *η* of DSSCs, which were obtained from four cells corresponding to each density of GNPs as shown in Additional file 1: Figure S1 (a) and (b), respectively, are considerably large. It is found that in each lot, *J*sc and *η* show the maximum values at GNP densities of 1.3 or 2.7 μg/cm^2^. Furthermore, the relation between *J*sc or *η* and the densities of GNPs in other experimental lots, in which GNP layers were formed at the interface between the conductive glass and TiO_2_ layers with various thicknesses, is shown in Additional file 2: Figure S2 (a) and (b), respectively. These results also show the similar tendency that *J*sc and *η* show the maximum values at GNP densities of 1.3 or 2.7 μg/cm^2^. However, the absolute values of *J*sc and *η* are smaller due to thinning of TiO_2_ layers. Therefore, GNPs with a density of 1.3 or 2.7 μg/cm^2^ are found to be optimum and were applied for investigation of the correlation between the position of a GNP layer in the TiO_2_ layer on the substrate and the optical absorption characteristics of TiO_2_ and the DSSC performance.

**Fig. 5:**
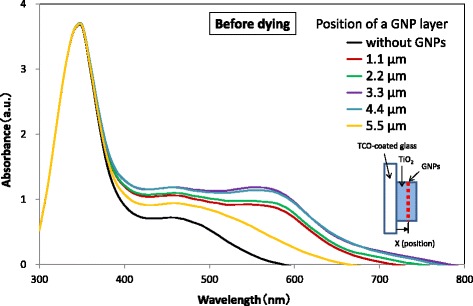
Absorbance spectra of TiO_2_ layers with varying the position of a GNP layer. The density of GNPs is 2.7 μg/cm^2^

**Fig. 6:**
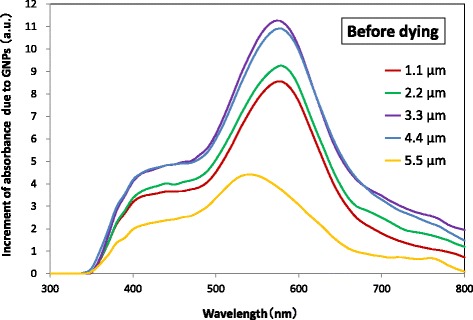
Increment of absorbance due to GNPs with varying the position of a GNP layer in the TiO_2_ layer. The density of GNPs is 2.7 μg/cm^2^

**Fig. 7:**
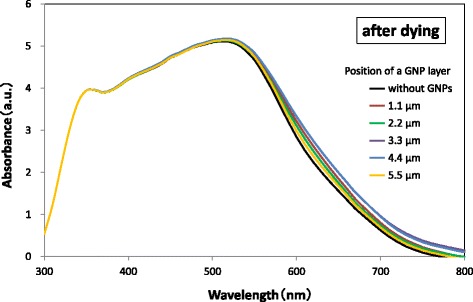
Absorbance spectra of TiO_2_ layers with varying the position of a GNP layer. The density of GNPs is 2.7 μg/cm^2^

The absorption spectra of TiO_2_ layers without and with a GNP layer deposited at various positions in the TiO_2_ layer without N719 dye are shown in Fig. 5. The position of a GNP layer was defined by the distance between the GNP layer and the TCO surface. The absorbance of a TiO_2_ layer with a GNP layer at any position was found to be larger than that of a TiO_2_ layer without a GNP layer. Figure 6 shows the increment of absorbance due to the application of GNPs, which was obtained by subtracting the absorbance of the TiO_2_ layer without GNPs from that of the TiO_2_ layer with GNPs shown in Fig. 5. It should be noted that the increment of the absorbance due to GNPs increases with increasing distance of the GNP layer from 1.1 to 3.3 μm or 4.4 μm from the TCO surface and then decreases with increasing distance from 4.4 to 5.5 μm, suggesting the distance that yields the maximum increment of the absorbance is around 4.0 μm. The increment can be observed in a wide wavelength range of 350–800 nm, but is particularly distinct in the range 500–650 nm. The absorption spectra of TiO_2_ layers without and with a GNP layer formed at various positions in the TiO_2_ layer sensitized with N719 dye are shown in Fig. 7. The absorption spectrum also shows a maximum at a distance of the GNP layer 3.3 or 4.4 μm (i.e., ~ 4.0 μm) from the TCO surface, suggesting that the absorption of N719 dye was enhanced effectively at this GNP layer position.

**Fig. 8:**
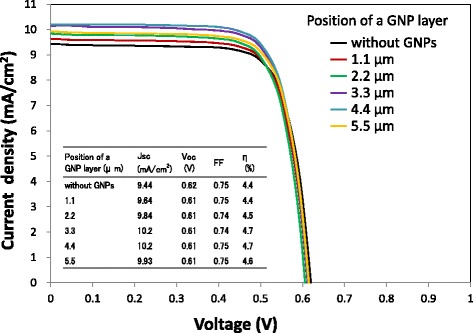
Current density–voltage curves of the DSSCs with varying the position of a GNP layer. The density of GNPs is 2.7 μg/cm^2^

**Fig. 9:**
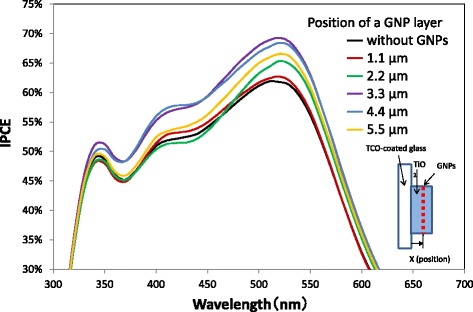
IPCE spectra of the DSSCs with varying the position of a GNP layer. The density of GNPs is 2.7 μg/cm^2^

**Fig. 10:**
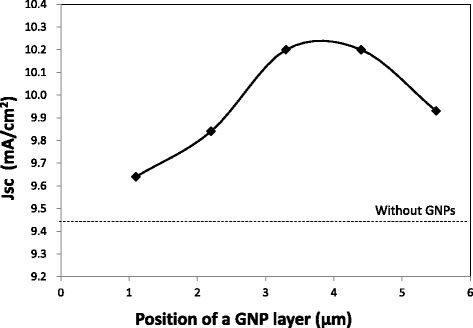
Dependence of *J*sc on the position of a GNP layer. The density of GNPs is 2.7 μg/cm^2^

**Fig. 11:**
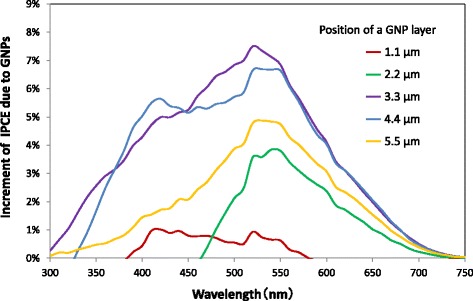
Increment of IPCE due to GNPs with varying the position of a GNP layer. The density of GNPs is 2.7 μg/cm^2^

Current density–voltage curves and IPCE spectra of the DSSCs with a GNP layer formed at various positions in the TiO_2_ layer are shown in Figs. 8 and 9, respectively. It is found that both current density and IPCE of DSSCs with a GNP layer formed at any position are larger than those of DSSCs without a GNP layer. The current density and IPCE with a GNP layer increase with increasing distance of the GNP layer from 1.1 to 3.3 μm or 4.4 μm (i.e., ~ 4.0 μm) and decrease with increasing distance to 5.5 μm. Figure 10 shows the dependence of *J*sc on the position of the GNP layer obtained from Fig. 8. Obviously, the maximum *J*sc was obtained when the GNP layer is ~ 4.0 μm from the TCO surface. It is found that the increase in *J*sc leads to an increase in *η*, as *V*oc and FF hardly change for all positions of the GNP layer, as shown in the inset table in Fig. 8. As the density of GNPs is the same for all GNP layers at any position, application of GNPs at ~ 4.0 μm from the TCO surface can be considered the most effective. By subtracting the IPCE of DSSCs without a GNP layer from that of DSSCs with a GNP layer shown in Fig. 9, the increment of IPCE owing to the application of GNPs was obtained, as shown in Fig. 11. We found that the increment of IPCE is the largest when the GNP layer exists at ~ 4.0 μm from the TCO surface. The increment can be seen in a wide wavelength range of 350–750 nm and becomes particularly large near 520 nm, showing a similar tendency to the absorption spectra in Fig. 6, suggesting that the increase in the IPCE is due to the enhancement of light absorption caused by the plasmon effects of GNPs.

**Fig. 12:**
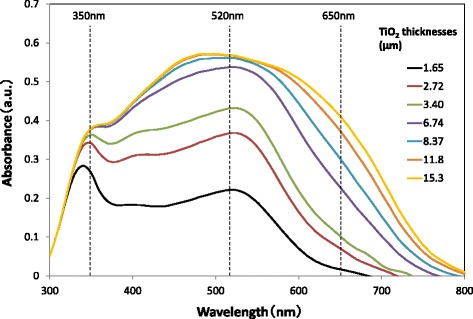
Absorbance spectra of dyed TiO_2_ layers with various thicknesses. The TiO_2_ layers do not contain GNPs

**Fig. 13:**
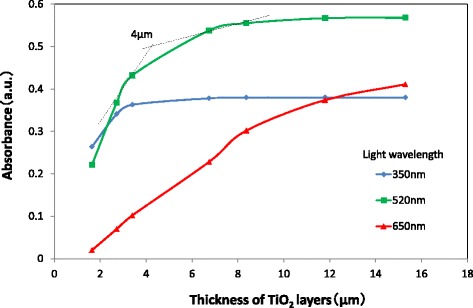
Correlation between light absorbance at various wavelengths and thicknesses of the TiO_2_ layers

Figure 12 shows the absorbance spectra of TiO_2_ layers of various thicknesses. Here, N719 dye is adsorbed and GNPs are not applied for all TiO_2_ layers. The absorbance is found to increase due to the increase in the quantity of adsorbed N719 dye with increasing TiO_2_ layer thickness. It is also found that the absorbance peaks near 520 nm of wavelength due to the light absorption of the dye. Therefore, the increment of IPCE by GNPs in Fig. 11 can be explained by enhancing the light absorption of N719 due to the plasmonic effect of GNPs. From Fig. 12, a correlation between the absorbance of light with the wavelengths of 350, 520, or 650 nm and the thickness of the TiO_2_ layer was obtained, as shown in Fig. 13. It is obvious that the absorbance of the TiO_2_ layer with light of a longer wavelength of 650 nm increases constantly with increasing TiO_2_ layer thickness. This means that the light of 650 nm penetrates the TiO_2_ layer deeper than 15.3 μm and is absorbed effectively. On the other hand, the absorbance of the TiO_2_ layer with light of a shorter wavelength of 350 nm saturates at a TiO_2_ layer thickness of ~ 3.0 μm, suggesting that the light of 350 nm is completely absorbed within ~ 3.0 μm of depth in the TiO_2_ layer. It should be noted that the absorbance saturates at a TiO_2_ layer thickness of ~ 4.0 μm for the light of 520 nm, which is the most effective in enhancing the performance of DSSCs due to the plasmonic effect of GNPs. The light with a wavelength of 520 nm can be considered almost fully absorbed by N719 dye in the TiO_2_ layer up to ~ 4.0 μm from the TCO surface and can hardly reach the position further than ~ 4.0 μm. Therefore, the enhancement in *J*sc decreases when the position of a GNP layer in the TiO_2_ layer becomes more than ~ 4.0 μm from the TCO surface as seen in Fig. 10 can be explained by a decrease in the absorption of light of 520 nm. On the other hand, the reason why the enhancement in *J*sc and light absorption of TiO_2_ layers increases as the distance of the GNP layer from the TCO surface becomes larger in the region of less than ~ 4.0 μm is not clear at this stage. However, when GNPs exist at ~ 4.0 μm from the TCO surface, which corresponds to the furthest distance of the light of 520 nm can reach in the TiO_2_ layer, light scattering by GNPs may have considerable contributions to the enhancement in DSSC performance by increasing the optical path length. The result of the dependence of DSSC performance on the position of the GNP layer suggests that GNPs existing at positions further than ~ 4.0 μm from the TCO surface are hardly useful for enhancing the light absorption of N719 dye, and thus are wasted in conventional DSSCs with metal nanoparticles distributed uniformly in the TiO_2_ layer. The penetration depth of the light of ~ 520 nm is ~ 4.0 μm in this study, but it may change depending on the quantity of adsorbed N719 dye and the intensity of the light irradiation.

### Enhancement of the Performance of DSSCs with Increasing the Number of Au Nanoparticle Layers

**Fig. 14:**
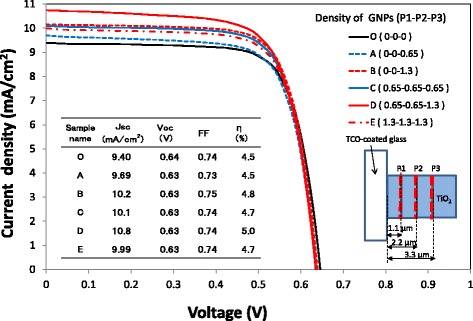
Current density–voltage curves of the DSSCs with varying the number of GNP layers and the density of GNP layers. The GNP layers of P1, P2, and P3 were formed at positions of 1.1, 2.2, and 3.3 μm from the TCO surface, respectively. The numbering in the legend with the format (P1-P2-P3) shows the density of GNPs (μg/cm^2^) at each position

**Fig. 15:**
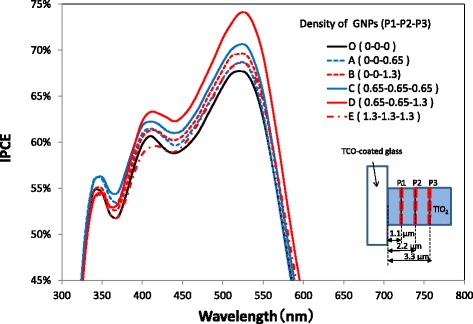
IPCE spectra of the DSSCs with varying the layer number and the density of GNPs. The GNP layers of P1, P2, and P3 were formed at positions of 1.1, 2.2, and 3.3 μm from the TCO surface, respectively. The numbering in the legend with the format (P1-P2-P3) shows the density of GNPs (μg/cm^2^) at each position

The irradiated light is scattered and absorbed on the surface of metal nanoparticles, and an evanescent light wave with a strong electromagnetic field is generated and localized on the surface of the nanoparticles. The evanescent light wave remains in the vicinity of the metal nanoparticle surface within a distance less than the diameter of the metal nanoparticle and the plasmon sensitivity decreases exponentially with distance away from the nanoparticle surface [32, 33]. Therefore, the light absorption of only N719 dye molecules located within ~ 40 nm from the surface of GNPs may be enhanced in this study, while the others are hardly affected, supporting the result that the increase in *J*sc is as large as 36% by applying a GNP layer to a thin TiO_2_ layer of 1.3 μm as shown in Table 1, but this increase becomes only 8.1% when applying a GNP layer to a thick TiO_2_ layer of 6.0 μm, as shown in Fig. 4. In an attempt to enhance the performance of DSSCs with a thick TiO_2_ layer, the number of GNP layers in the TiO_2_ layer was increased. Current density–voltage curves and IPCE spectra of DSSCs with varying the number of GNP layers and the density of GNPs are shown in Figs. 14 and 15, respectively. Three levels of GNP layers named P1, P2, and P3 are shown in the inset of Fig. 14, which were formed at positions of 1.1, 2.2, and 3.3 μm, respectively, from the TCO surface. The current densities and IPCEs of the DSSCs (A–E) with a GNP layer formed at the position of P3 in the TiO_2_ layer are found to be larger than those of the DSSC (O) without a GNP layer. Moreover, the performance of the DSSC (B) with a GNP density of 1.3 μg/cm^2^ is found to be better than that of the DSSC (A) with a GNP density of 0.65 μg/cm^2^. It should be noted that the addition of GNP layers with a GNP density of 0.65 μg/cm^2^ to the positions of P1 and P2, which are located near the front of the incident irradiation, improves *J*sc more significantly. However, increases in *J*sc were not observed by adding GNP layers with a GNP density of 1.3 μg/cm^2^ to the positions of P1 and P2 (E). The reason why the large quantity of GNPs existing near the front of the incident irradiation decreases *J*sc is unknown; however, it is speculated that some of these GNPs may aggregate and affect the absorption of GNPs at P3 by scattering the incident irradiation, judging from the SEM observation that GNPs aggregate in some parts of the TiO_2_ layers. The DSSC (D), in which three levels of the GNP layer with a GNP density of 0.65, 0.65, and 1.3 μg/cm^2^, were formed at positions of P1, P2, and P3, respectively, shows the best performance with *J*sc and *η* of 10.8 mA/cm^2^ and 5.0%, increases of 15 and 11%, respectively, compared with those of the DSSCs without a GNP layer. In other words, the best performance was obtained when relatively high concentrations of GNPs were formed at the position which is the penetration depth of the incident light of the wavelength corresponding to the maximum absorption of N719 dye (~ 520 nm) and relatively low concentrations of GNPs were formed in the path of the incident light before this position. Nevertheless, the increase in the performance of these DSSCs is not high enough compared with that of DSSCs with a thin TiO_2_ layer. In this study, TiO_2_ paste was applied by a screen-printing method, with which the limit of the thinnest a TiO_2_ layer was ~ 1 μm after annealing, owing to the requirement of uniformity and reproducibility of its thickness. The thickness is considered too large to obtain a higher plasmonic enhancement. A spraying method using TiO_2_ paste diluted with a solvent may be useful for reproducibly obtaining thinner TiO_2_ layers. Increasing the ratio of GNP layers to TiO_2_ layers with the technology of fabricating very thin TiO_2_ layers may further enhance the performance of DSSCs. In addition, ~ 40 nm GNPs were used in the present study to reduce variations in GNP size, but with ~ 60 nm GNPs, there is a possibility that the performance may be further improved, judging from Table 1. Changing the size of GNPs at each GNP layer formed in the TiO_2_ may improve the DSSC performance even more. It has been reported that the ratio of plasmon scattering to absorption increases with increasing volume of GNPs [34]. Formation of large GNPs near the back of the optical path through the TiO_2_ layer may improve DSSC performance due to prolonging the optical path length by light scattering. Although the distribution of GNPs and the thickness of a TiO_2_ layer have not yet been optimized, the purpose of this study, which was to confirm whether the performance of DSSCs can be improved by optimizing the distribution of GNPs for plasmonic enhancement, has been achieved.

## Conclusions

The dependence of the light absorption and the performance of DSSCs on the position of a GNP layer in the TiO_2_ layer was investigated. The absorption of the TiO_2_ layer and the performance of the DSSC are increased the most by the plasmonic enhancement when GNPs are concentrated near the position in the TiO_2_ layer which is the penetration depth of the incident light of wavelength corresponding to the maximum absorption of N719 dye (~ 520 nm). The performance of DSSCs is found to be improved more by adding GNP layers with relatively low concentrations of GNPs near the front of the incident irradiation. *J*sc and *η* of the DSSC with three levels of the GNP layer applied in the TiO_2_ layer were 10.8 mA/cm^2^ and 5.0%, increases of 15 and 11%, respectively, compared with those of the DSSCs without a GNP layer. Optimization of the distribution of GNPs in the TiO_2_ layer has been found to be very important for improving the performance of DSSCs employing GNPs.

## Additional Files

Additional file 1: Figure S1. (a) *J*sc and (b) *η* of the DSSCs with varying density of GNPs. The thickness of TiO_2_ layer is 6.0 μm. (PDF 274 kb)

Additional file 2: Figure S2. (a) *J*sc and (b) *η* of the DSSCs with varying the density of GNPs. GNP layers were formed at the interface between the conductive glass and TiO_2_ layers of 1.4 μm (3rd and 4th lots) and 1.8 μm (5th lot) thicknesses, respectively. (PDF 278 kb)

## Abbreviations

DSSC: Dye-sensitized solar cells
FF: Fill factor
GNPs: Au nanoparticles
IPCE: Incident photon-to-current efficiency
ITO: Indium tin oxide
*J*sc: Short-circuit current density
*J–V*: Current density–voltage
N719: Di-tetrabutylammonium *cis*-bis (isothiocyanato) bis (2, 2-bipyridyl-4, 4′-dicarboxylato) ruthenium (II)
SEM: Scanning electron microscope
TBP: 4-Tert-butylpyridine
TCO: Transparent conductive oxide
TEM: Transmission electron microscope
*V*oc: Open-circuit voltage
*η*: Energy conversion efficiency
