# Supplementary figures and images for: Effect of Gold Nanoparticle Distribution in TiO2 on the Optical and Electrical Characteristics of Dye-Sensitized Solar Cells
Source: Nanoscale Res Lett. 2017 Aug 29;12:513. doi: 10.1186/s11671-017-2285-4 (PMC5574828; doi:10.1186/s11671-017-2285-4)

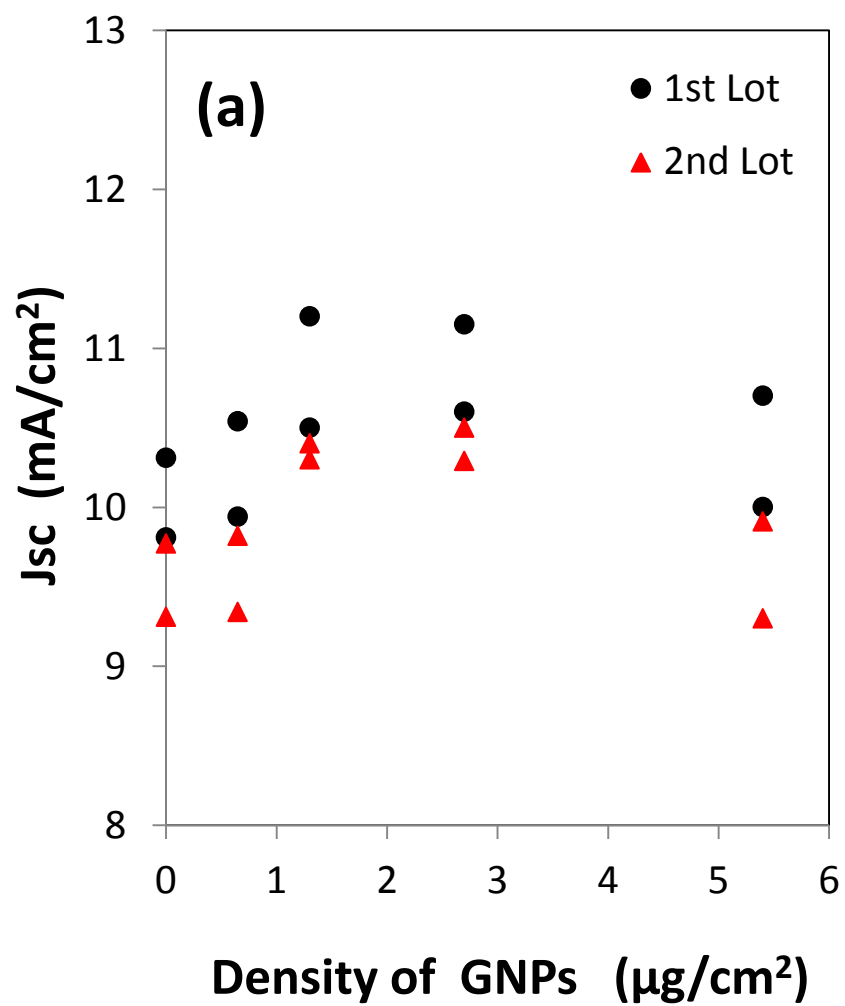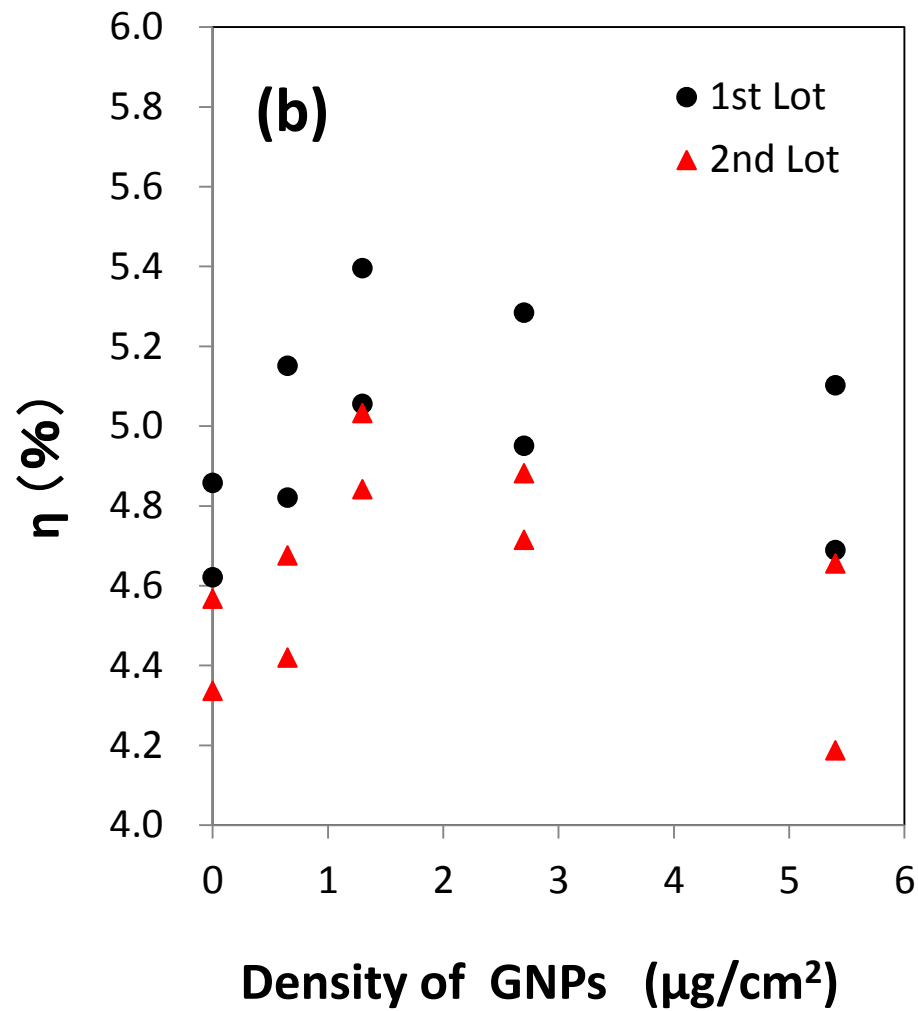

**Figure S1.**

Supplement: Supplementary file 1 — (a) Jsc and (b) η of the DSSCs with varying density of GNPs. The thickness of TiO2 layer is 6.0 μm. (PDF 274 kb) [file 11671_2017_2285_MOESM1_ESM.pdf]

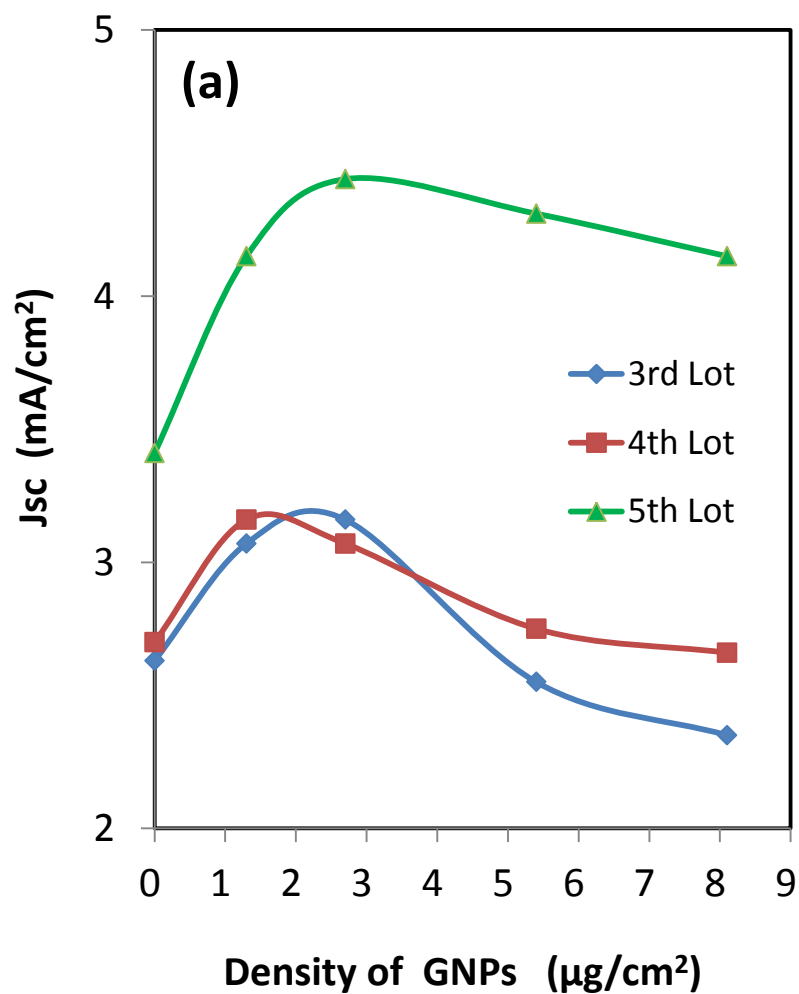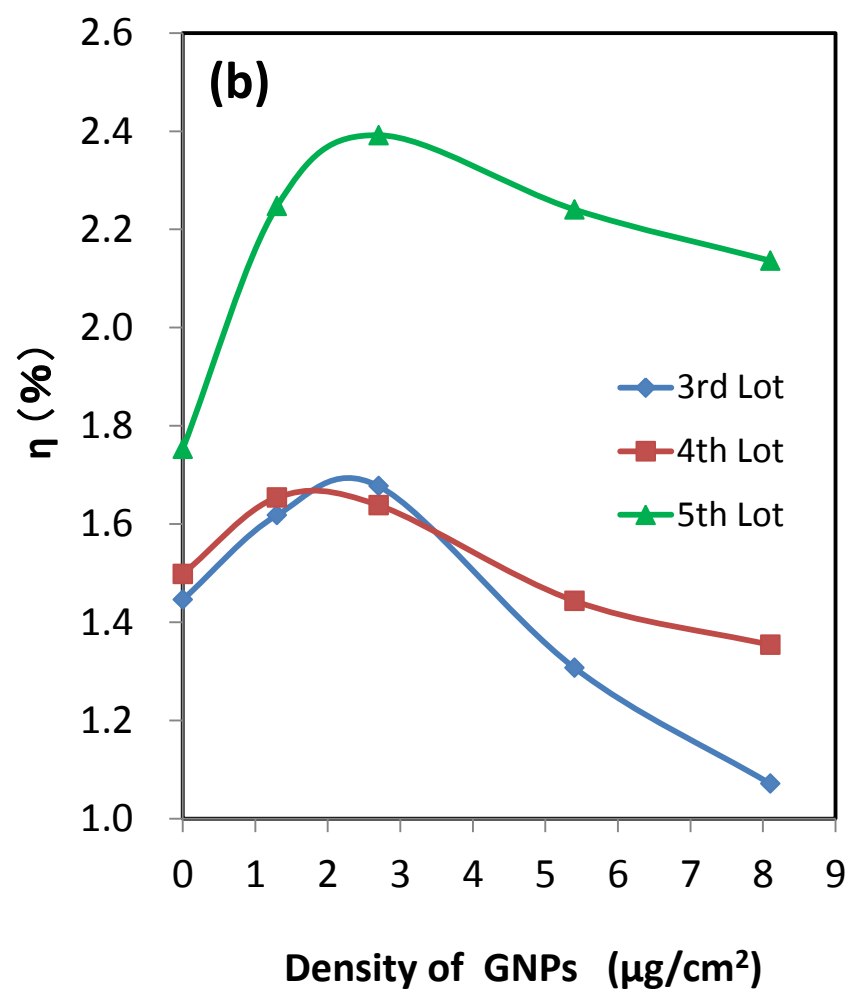

**Figure S2.**

Supplement: Supplementary file 2 — (a) Jsc and (b) η of the DSSCs with varying the density of GNPs. GNP layers were formed at the interface between the conductive glass and TiO2 layers of 1.4 μm (3rd and 4th lots) and 1.8 μm (5th lot) thicknesses, respectively. (PDF 278 kb) [file 11671_2017_2285_MOESM2_ESM.pdf]
